# Methanolic extract of *Justicia secunda* ameliorates the cyclophosphamide-induced hepatic and renal failures in rats

**DOI:** 10.22038/ajp.2024.25237

**Published:** 2025

**Authors:** Winner Oyidiya Kalu, Chinedum Ogbonnaya Eleazu, Ngozi Kalu Achi, Mercy Amarachi Iroaganachi, Duru Majesty

**Affiliations:** 1 Department of Biochemistry, Rhema University, Aba, Abia State, Nigeria; 2 Department of Biochemistry, Alex Ekwueme Federal University, Ndufu-Alike, Ebonyi State, Nigeria; 3 Department of Biochemistry, Michael Okpara University of Agriculture, Umudike, Umuahia, Abia State, Nigeria; 4 Abia State Polytechnic, Aba, Abia State, Nigeria

**Keywords:** Natural products, Antioxidants, Chemotherapeutic agents, Oxidative stress, Nutraceuticals

## Abstract

**Objective:**

This study determined the effect of the methanolic extract of *Justicia secunda* against cyclophosphamide-instigated hepatic and renal toxicities in rats and analyzed the bioactive constituents of the extract using gas chromatography mass spectrophotometry (GC-MS).

**Materials and Methods:**

Twenty male albino Wistar rats were assigned into four groups of five rats each: Group 1 received rat feeds and tap water for 14 days. Group 2 received rat feeds and water for 14 days and cyclophosphamide (CPH, 100 mg/kg.BW) on day 15. Group 3 received rat feeds and 200 mg/kg.BW of the extract for 14 days and CPH on day 15 while Group 4 received rat feeds and 400 mg/kg.BW of the extract for 14 days and CPH on day 15.

**Results:**

CPH induction altered the final body weights, hepatic and renal total proteins, antioxidant markers, liver and kidney weights, and serum transaminases, urea, and creatinine concentrations of the rats, inducing lipid peroxidation in them which was mitigated following supplementation with *J. secunda*. GC-MS assay showed the presence of twenty compounds in *J. secunda* extract and acute toxicity study (using mice to determine the safety profile of the extract) showed the safety of the usage of the plant at 200 and 400 mg/kg doses.

**Conclusion:**

*J. secunda *has protective effects against CPH-induced hepatic and renal toxicities which could be attributed to its bioactive compounds.

## Introduction

The use of chemotherapeutic agents to treat cancer has increased the chances of cancer survival (Curigliano et al., 2016). Although efficacious in cancer treatment, these chemotherapeutic agents do not differentiate between cancerous and normal cells thereby causing systemic and organ toxicities to cancer patients such as cardiotoxicity, neurotoxicity, nephrotoxicity, hepatotoxicity, etc (Khan et al., 2013). This has therefore become a problem of utmost concern to clinicians (Ekeleme-Egedigwe et al., 2019). 

Cyclophosphamide (CPH) is an alkylating agent that is generally used as an anticancer agent in cancer chemotherapy as well as an immunosuppressive agent (Mansour et al., 2017; Mahmoud et al., 2017).

CPH is metabolically activated by the hepatic cytochrome-P450 enzymes and its activation generates acrolein and phosphoramide as its metabolites. Phosphoramide is responsible for the anticancer and immunosuppressive action of CPH, while acrolein, which is known to be very reactive with a short biological half-life, interferes with the antioxidant defense mechanism of the body, inducing the generation of reactive oxygen species (ROS) (Ekeleme-Egedigwe et al., 2019; Shokrzadeh et al., 2017; Moghe et al., 2015). The increased ROS production leads to a state of oxidative stress with resultant damage to tissues (Kratchanova et al., 2010). Therefore, CPH metabolite, acrolein is suggested to be behind the adverse effects of CPH such as hepatotoxicity, renal toxicity, reproductive toxicity, cardiotoxicity, bone marrow suppression, etc. (Ekeleme-Egedigwe et al., 2019; Moghe et al., 2015; Raza and Alghasham, 2011; Habibi et al., 2015; Omole et al., 2018; Arena et al., 2018; Taslimi et al., 2019). 

Plants have increasingly been used worldwide in the treatment of several human diseases in recent times. This is because, they serve as important sources of some antioxidant phytochemicals that protect the human body from free radical-associated diseases, in addition to possessing health promoting properties (Eleazu et al., 2011; Eleazu et al., 2014a; Iqbal et al., 2015).

*Justicia secunda* Vahl. which belongs to the family of Acanthaceae, is commonly known as “blood root” in Barbados whereas in Venzuela, it is known as “sanguinaria” (Onoja et al., 2017). In the South-Eastern part of Nigeria, its local name is “obara bundu”. The plant grows in humid soils around rivers or creeks and it can be found in tropical and pantropical regions of the world (Herrera-Mata et al., 2002). Some of the biological properties that have been credited to *J. secunda* include antioxidant, anti-inflammatory, antinociceptive, and hepatoprotective properties (Onoja et al., 2017; Anyasor et al., 2020).

Despite the array of pharmacological benefits of *J. secunda *as reported above, the possibility of its protective action against CPH-induced hepatotoxicity and renal toxicity has not been explored. In the search for natural remedies that can attenuate the adverse effects of CPH but preserve its anticancer potentials and considering the antioxidant properties of *J. secunda*, the present study explored the protective action of methanol extract of *J. secunda* against CPH-instigated hepatic and renal oxidative stress and elevation of hepatic and renal function markers in rats. The bioactive components of the extract were also analyzed using gas chromatography mass spectrophotometry (GC-MS).

## Materials and Methods

### Chemicals and reagents

CPH that was used for this study was bought from the Sigma and Aldrich Chemical Company (St Louis, MO, USA). The urea and creatinine kits were obtained from Randox Laboratory Limited (Alexandre, London, UK). Additional chemicals that we used for this study, which are not mentioned here, were of the highest grade. 

### Plant collection and identification

Fresh leaves of *J. secunda* were collected from a farmland in Aba, Abia State, Nigeria. Authentication of the leaves was carried out at the Plant Science and Biotechnology Department, Michael Okpara University of Agriculture, Umudike, Nigeria.

### Preparation of J. secunda extract

The leaves were subjected to air drying at room temperature, after which, they were ground to flour with a manual milling machine (Corona, China). A weighed quantity (360 g) of the flour was soaked in 1 L of 80% methanol. The mixture was shaken every 3 hr and allowed to stand for 48 hr at room temperature. Thereafter, it was filtered with Whatman No.1 filter paper and the filtrate was concentrated (at 40^o^C) in an oven until all the methanol was evaporated. The extract yield was calculated and is reported as a percentage. The extract was thereafter, stored in a refrigerator until usage (Onoja et al., 2017; Akah et al., 2009). The weight of the extract was 17.6 g while the yield (in percentage) was 4.88%. 

### Experimental animals

Male Wistar rats with an average weight of 170±10 g, were bought from the animal house of the Faculty of Veterinary Medicine, University of Port-Harcourt, Rivers State, Nigeria. Acclimatization of the animals was done for two weeks under standard environmental conditions and they received their feed (vital feed) and water *ad libitum*. Ethical approval to conduct this study was sought and obtained from the Rhema University’s Committee on the care and use of laboratory animals, and the study was conducted following the revised guideline for the care and use of laboratory animals as reported by the National Institute of Health (Pub No. 85-23, revised 1985).

### Acute toxicity test

Thirty-five mice (males and females) weighing 20-25 g were classified into 7 groups of 5 mice per group and they were given different doses of methanol extract of *J. secunda *(i.p.) in the sequence: 500, 1000, 1500, 2000, 3000, 4000 and 5000 mg/kg.BW respectively. The mice were kept in aluminum cages and allowed free access to feed and water *ad libitum*. The mice were observed for twenty-four hours for mortality and signs of toxicity. The LD_50_ of the extract was then calculated using Karber’s method (Enigide et al., 2013) as follows: LD_50_ = LD_100_ - Σ ({D x M}/N). Where LD_50_ = The dose that killed fifty percent of the mice in a group. LD_100_ = The dose that killed all the animals in a group; D = Dose difference; M = Mean death; N = Number of mice in each group. 

The LD_50_ of the extract was obtained as approximately 4000 mg/kg.BW in mice. Following conversion to rats equivalent dose (which we obtained as 2000 mg/kg. BW) (Freireich et al., 1966), the doses of 200 and 400 mg/kg.BW (as intermediate and upper doses) were selected for further studies based on the OECD guideline (OECD, 2008). The doses of CPH used in this study were chosen based on a previous study (Parandin et al., 2023).

### Experimental design

Twenty male albino Wistar rats were acclimated for two weeks and thereafter, they were assigned into four groups of five rats per group as follows: 

Group 1: Control group that received standard rat feeds and tap water for 14 days. Group 2: CYP group that received rat feeds and water for 14 days and 100 mg/kg.BW CPH on day 15 (intraperitoneally, i.p.). Group 3: Study group 1 that received rat feeds and 200 mg/kg.BW *J. secunda* extract for 14 days and 100 mg/kg.BW CPH on day 15 (i.p.). Group 4: Study group 2 that received rat feeds and 400 mg/kg.BW *J. secunda* extract for 14 days and 100 mg/kg.BW CPH on day 15 (i.p.). 

Water was made available *ad libitum* to all the groups throughout the duration of the experiment. The body weight of each rat was taken every week. At the end of the treatment period, blood was collected from their retro-orbital veins and the rats were sacrificed under anesthesia (using chloroform). To harvest the serum of each rats, their blood samples were centrifuged for 10 min at 3,000 g and the sera were assayed for aspartate amino transaminase (AST), alanine amino transaminase (ALT), urea and creatine concentrations of the rats. The rats’ kidney and liver tissues were collected, weighed and grinded. The homogenates were assayed for superoxide dismutase (SOD), glutathione peroxidase (GPx), catalase (CAT), reduced glutathione (GSH), glutathione-S-transferase (GST), lipid peroxidation marker and total proteins. 

### Evaluation of hepatic and renal function markers

AST and ALT activities were analyzed following the procedure of Reitman and Frankel (1957). The concentrations of urea and creatinine were quantified using Randox Laboratory assay kits (London, UK). 

### Estimation of oxidative stress and antioxidant markers in the liver and kidney

GST activity was determined using the method of Habig et al (1972). GSH was determined using the method of Beutler et al. (1963). GPx activity was determined using the method of Paglia and Valentine (1967). SOD activity was determined using the method of Misra and Fridovich (1972). CAT activity was determined using the method of Sinha (1972). To analyze for lipid peroxidation, thiobarbituric acid reactive substances (TBARS) were quantified in the sera using the method of Varshney and Kale (1990). 

### Determination of total proteins in the liver and kidney

Assay for total proteins in the liver and kidney homogenates was carried out using Randox assay kits using the procedure of Tietz (1995). 

### GC-MS analysis 

The GC-MS assay to determine the bioactive compounds that are present in the *J. secunda* extract was done following the protocol of Ganesh and Mohankumar (2017) as modified by Iroaganachi et al (2023). The extract was constituted in the 80% methanol, filtered (0.22 µm nylon filter) and analyzed by GC-MS. To carry out the GCMS assay, an electron ionization system with ionizing energy of 70 eV was used while helium was the carrier gas. The flow rate was 1 ml/min, the injection volume of the extract was 2 µl while the split ratio was 1:75. The temperature of the injector was 250^o^C. The total time of the GC run was 40 min. Calculation of the percentage of each compound was done by comparing its average peak area to the total areas. 

### Identification of compounds

Identification of the bioactive compounds in the extract was done by matching the peaks of the extract with that from the computer library and the identity of the compounds was confirmed by comparing the mass spectra of the peaks with known spectra that were obtained from the database of the National Institute’s Standard and Technology’s data base (Ganesh and Mohankumar, 2017).

### Statistical analysis

Statistical analysis was carried out on the data that were generated, using version 21 of the Statistical Package for Social Sciences (SPSS). Data is presented as means±SD. Comparison of means was done using the One-way analysis of variance (ANOVA). Statistical significance level was set at p<0.05.

## Results

The data on the body weights and weight gain of the rats we investigated are presented in Table 1. No significant change (p>0.05) was obtained in the initial body weights of all the rats across the groups. However, at the end of the study, rats exposed to CPH alone had significant (p<0.05) decreases in their final body weights and weight gain compared to the control. On the other hand, rats in study groups 1 and 2 had significantly (p<0.05) increased final body weights and weight gain compared to the CYP group. 

**Table 1:** Initial and final body weight for rats (g) and weight gain

**Groups**	**Initial weight**	**Final weight**	**Weight gain (g)**
Control	178.60±10.82	198.76±7.36	18.59±1.37
CYP	176.63± 6.48	179.32±2.78^a ^	2.51±0.92^a ^
Study group 1	179.45± 9.36	197.88±9.74^b^	17.20±1.63^b ^
Study group 2	175.84 ±7.91	193.65±5.83^b^	17.88±1.20^b ^

Values are reported as means ± SD. ^a^p<0.05 in comparison with the control; ^b^p<0.05 in comparison with the cyclophosphamide (CYP) group. Study group 1 (CPH + 200 mg/kg.BW *J. secunda*); and Study group 2 (CPH + 400 mg/kg.BW *J. secunda*).

**Figure 1:**
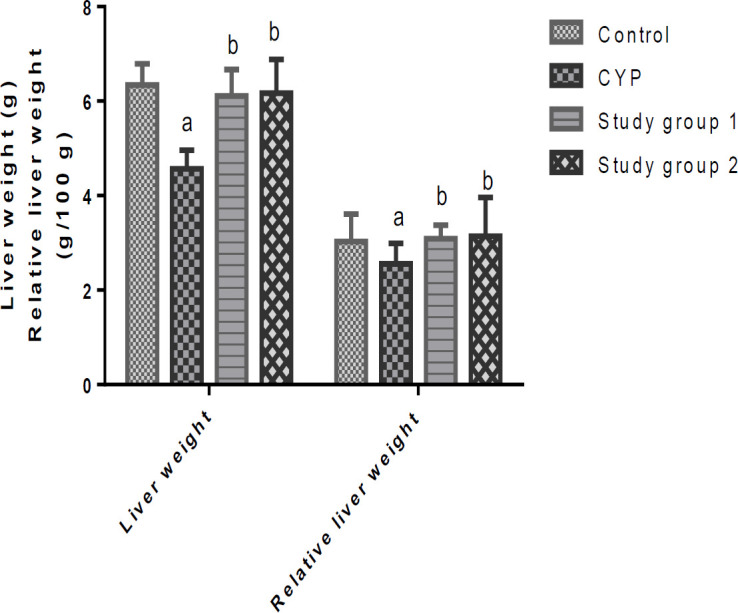
Liver weight and relative liver weight of rats. ^a^p<0.05 in comparison with the control; ^b^p<0.05 in comparison with the cyclophosphamide (CYP) group. Study group 1 (CPH + 200 mg/kg.BW J. secunda) and Study group 2 (CPH + 400 mg/kg.BW J. secunda).

**Figure 2:**
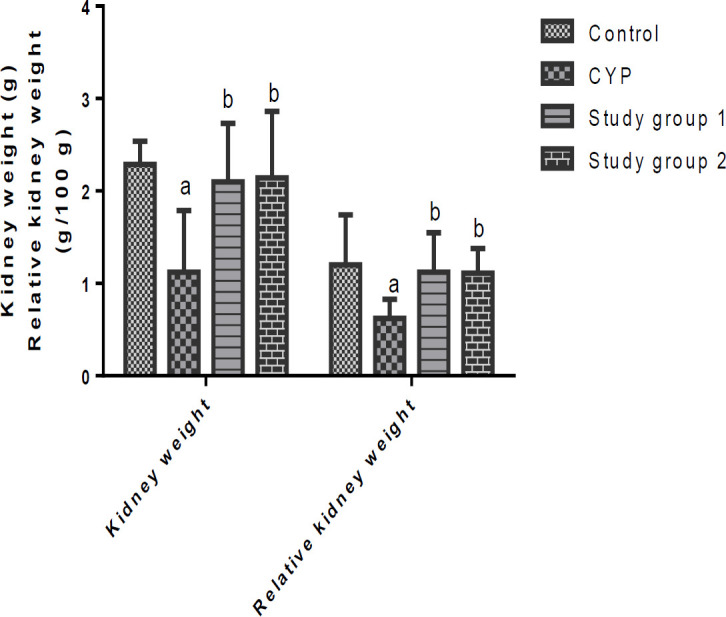
Kidney weight and relative kidney weight of rats. ^a^p<0.05 in comparison with the control; ^b^p<0.05 in comparison with the cyclophosphamide (CYP) group. Study group 1 (CPH + 200 mg/kg.BW J. secunda) and Study group 2 (CPH + 400 mg/kg.BW J. secunda).

The data on the weights of the organs and the relative weights of the organs of the rats that were investigated are shown in Figures 1 and 2. When compared to the control, the CYP group had significant (p<0.05) decreases in their liver and kidney weights as well as their relative liver and kidney weights. In contrast, supplementation with *J. secunda *resulted in significant (p<0.05) increases in the liver and kidney weights and the relative liver and kidney weights of the rats in study groups 1 and 2, relative to the CYP group. 

The effects of *J. secunda* on the liver and kidney function markers in the sera of the studied rats are presented in Table 2. As presented in the Table, there were marked elevations (p<0.05) in the activities of the liver function markers (ALT and AST) and the concentrations of the kidney function markers (urea and creatinine) in the sera of the CYP group compared to the control. In contrast, significant decreases (p<0.05) were obtained in the serum ALT and AST activities as well as the serum urea and creatinine concentrations of study groups 1 and 2, relative to the CYP group. Further, no significant change (p>0.05) was found in the serum urea concentrations of study groups 1 and 2, relative to the control. 

The effects of *J. secunda* on the oxidative stress and antioxidant markers in the liver and kidneys of the rats that we investigated are presented in Tables 3 and 4. Compared with the control, the liver and renal activities of GST, GPx, SOD and CAT in the CYP group as well as their liver and renal GSH concentrations were markedly decreased (p<0.05). On the contrary, the liver and renal GST, GPx, SOD and CAT activities of the rats in study groups 1 and 2 and their liver and renal GSH concentrations were markedly (p<0.05) elevated relative to the CYP group. Furthermore, no significant difference (p>0.05) was found between the liver and renal GST and CAT activities of study group 2 rats and the control, and between the renal GPx activities of study groups 1 and 2 and the control. 

**Table 2:** Hepatic and renal function bio-markers in the sera of rats.

**Groups**	**ALT (U/L)**	**AST (U/L)**	**Urea (mg/dl)**	**Creatinine (mg/dl)**
Control	53.18±8.61	45.32±10.11	69.63±6.91	0.78±0.20
CYP	81.68±11.47^a^	68.56± 5.02^a^	90.32±4.58^a^	1.89± 0.17^a^
Study group 1	69.28±14.19^b^	52.09±7.53^b^	71.85± 7.11^b,c^	1.18±0.95^b^
Study group 2	65.70±6.30^b^	49.54±11.71^b^	67.47±10.54^b,c^	1.66± 0.64^b^

Values are reported as means ± SD. ^a^p<0.05 in comparison with the control; ^b^p<0.05 in comparison with the cyclophosphamide (CYP) group; ^c^P>0.05 in comparison with the control. Study group 1 (CPH + 200 mg/kg.BW *J. secunda*) and Study group 2 (CPH + 400 mg/kg.BW *J. secunda*). ALT: Alanine amino transaminase; and AST: Aspartate amino transaminase

**Table 3:** Oxidative stress and antioxidant indices in the liver of rats.

**Groups**	**GST**	**GSH**	**GPx**	**SOD**	**CAT**	**LPO**
Control	9.15±1.23^c^	5.53±0.19	3.17±0.27	0.86±0.04	2.71±0.53	0.62±0.27
CYP	2.45±0.38^a^	1.07±0.50^a^	0.57±0.11^a^	0.14±0.06^a^	0.66±0.41^a^	2.48±0.18^a^
Study group 1	6.76±0.67^b^	3.40±0.08^b^	1.92±0.33^b^	0.75±0.12^b^	2.22±0.69^b^	1.62±0.53^b^
Study group 2	8.58±0.95^b^	4.93±0.72^b^	2.86±0.54^b^	0.81±0.15^b^	2.49±0.38^b^	1.34±0.36^b^

Values are reported as means ± SD. ^a^p<0.05 in comparison with the control; ^b^p<0.05 in comparison with the cyclophosphamide (CYP) group. Study group 1 (CPH + 200 mg/kg.BW *J. secunda*) and Study group 2 (CPH + 400 mg/kg.BW *J. secunda*). GST (Glutathione-S-transferase)- µ mole CDNB-GSH/min/mg protein; GSH (reduced glutathione)- µmole/mg; GPx (Glutathione peroxidase)- units/mg protein; SOD (Superoxide dismutase)- µmole epinephrine oxidized/min/mg protein; CAT (Catalase)- nmole H_2_O_2 _consumed/min/mg protein; and LPO (Lipid peroxidation)- µmole MDA formed/mg protein.

**Table 4:** Oxidative stress and antioxidant indices in the kidney of rats.

**Groups**	**GST**	**GSH**	**GPx**	**SOD**	**CAT**	**LPO**
Control	7.42±1.65	4.48±1.66^c^	2.98±0.51	0.61±0.23	2.86±1.28	0.51± 0.67
CYP	2.14±0.77^a^	1.29±0.25^a^	0.63±0.19^a^	0.11±0.09^a^	0.78 ± 0.11^a^	2.94± 0.35^a^
Study group 1	5.25±1.05^b^	2.11±0.59^b^	2.57±0.86^b,c^	0.43±0.17^b^	1.92± 0.20^b^	1.78±0.08^b^
Study group 2	6.93±0.89^b,c^	4.87±0.72^c^	2.64±0.45^b,c^	0.49±0.05^b^	2.34± 0.54^b,c^	1.10±0.11^b^

Values are reported as means ± SD. ^a^p<0.05 in comparison with the control; ^b^p<0.05 in comparison with the cyclophosphamide (CYP) group; ^c^p>0.05 in comparison with the control; Study group 1 (CPH + 200 mg/kg.BW *J. secunda*) and Study group 2 (CPH + 400 mg/kg.BW *J. secunda*). GST (Glutathione-S-transferase)- µ mole CDNB-GSH/min/mg protein; GSH (reduced glutathione)- µmole/mg; GPx (Glutathione peroxidase)- units/mg protein; SOD (Superoxide dismutase)- µmole epinephrine oxidized/min/mg protein; CAT (Catalase)- nmole H_2_O_2 _consumed/min/mg protein; and LPO (Lipid peroxidation)- µmole MDA formed/mg protein.

MDA (a marker of lipid peroxidation) concentration was markedly elevated (p<0.05) in the liver and kidney of the CYP group compared with the control. On the contrary, the MDA concentrations in the liver and kidneys of study groups 1 and 2 rats were significantly decreased (p<0.05) when compared with the CYP group. 

**Figure 3:**
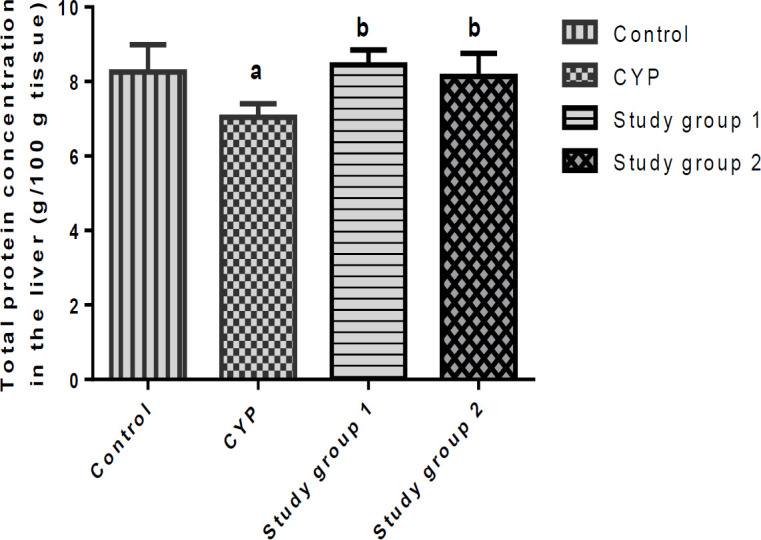
Total protein contents in the liver of rats. ^a^p<0.05 in comparison with the control; ^b^p<0.05 in comparison with the cyclophosphamide (CYP) group. Study group 1 (CPH + 200 mg/kg.BW J. secunda); Study group 2 (CPH + 400 mg/kg.BW J. secunda)

**Figure 4:**
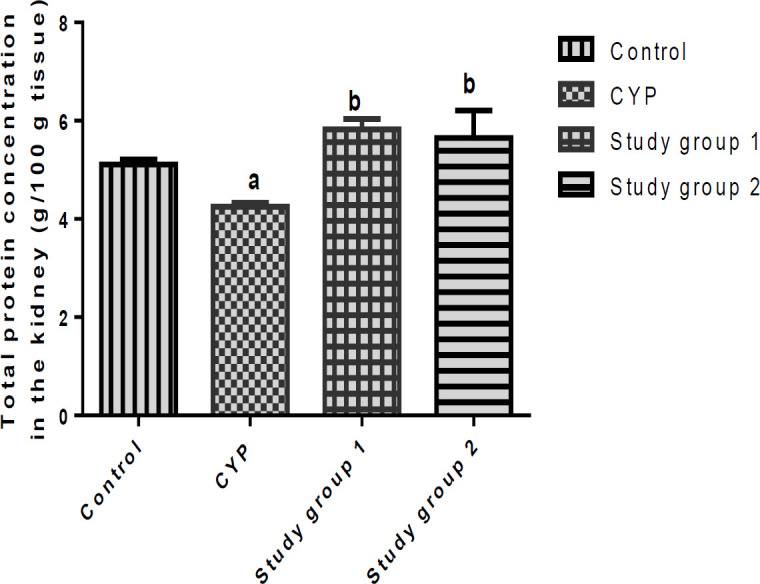
Total protein contents in the kidneys of rats. ^a^p<0.05 in comparison with the control; ^b^p<0.05 in comparison with the cyclophosphamide (CYP) group. Study group 1 (CPH + 200 mg/kg.BW J. secunda) and Study group 2 (CPH + 400 mg/kg.BW J. secunda).

The effects of *J. secunda* on the total protein concentrations in the liver and kidneys of the investigated rats are presented in Figures 3 and 4. Data shown in the Figures reveals a significant decline (p<0.05) in the concentrations of total protein in the liver and kidneys of the CYP group compared with the control, but significant increases (p<0.05) in the concentrations of total proteins in the liver and kidneys of the rats in study groups 1 and 2, when compared to the CYP group. 

Results of the GC-MS analysis of *J. secunda* indicated the presence of twenty compounds (compounds and their retention times (RT) are shown in Table 5). Table 6 summarizes the compounds in the extract with identified biological activities and their classes. 

**Table 5:** Results of GC-MS analysis of J. secunda Vahl. leaf extract

	**Retention Time**	**Name of Compound **	**Molecular Formula**	**Molecular Weight**	**Peak Area%**
1	3.655	p-Xylene	C_8_H_10_	106	2.351
2	4.491	Desulphosinigrin	C_10_H_17_NO_6_S	279	2.420
3	5.114	Tetradecane, 2,6,10-trimethyl	C_17_H_36_	240	0.709
4	5.257	7-epi-cis-sesquisabinene hydrate	C_15_H_26_O	222	0.779
5	5.297	7-Ethyl-4-decen-6-one	C_12_H_22_O	182	1.587
6	5.435	Tridecane	C_13_H_28_	184	1.717
7	5.732	2-Cyclohexylpiperidine	C_11_H_21_N	167	1.199
8	6.493	10-Acetoxy-2-hydroxy-1,2,6a,6b,9,9,12a-heptamethyl 1,3,4,5,6,6a,6b,7,8,8a,9,10,11,12,12a,12b,13,14b -octadecahydro-2H-picene-4a-carboxylic acid, methyl ester	C_33_H_52_O_5_	528	0.925
9	6.539	1-Heptatriacotanol	C_37_H_76_O	536	0.790
10	6.722	17-Pentatriacontene	C_35_H_70_	490	1.241
11	6.814	Phytol, acetate	C_22_H_42_O_2_	338	13.179
12	6.871	Ethanol, 2-(9-octadecenyloxy)-, (Z)-	C_20_H_40_O_2_	312	3.914
13	6.917	3,7,11,15-Tetramethyl-2-hexadecen-1-ol	C_20_H_40_O	296	6.979
14	7.014	Pentadecanoic acid, 13-methyl-, methyl ester	C_17_H_34_O_2_	270	3.238
15	7.100	Hexadecanoic acid, 1-(hydroxymethyl)-1,2-ethanediyl ester	C_35_H_68_O_5_	568	3.037
16	7.495	Isophytol, acetate	C_22_H_42_O_2_	338	35.067
17	7.580	9,12,15-Octadecatrienoic acid, 2-phenyl-1,3-dioxan-5-yl ester	C_28_H_40_O_4_	440	3.907
18	8.336	Benzyl butyl phthalate	C_19_H_20_O_4_	312	2.579
19	9.034	Diisooctyl phthalate	C_24_H_38_O_4_	390	5.381
20	10.911	Squalene	C_30_H_50_	410	9.001

**Table 6:** Identified compounds in the extract, their biological activities and their classes

S/No	Compound Name	Class of Compound	Biological Activity
1	p-xylene	Aromatic hydrocarbon	Anti-cancer activity (Manzoor et al., 2016))
2	7-epi-cis-sesquisabinene hydrate	Terpene	Anti-cancer property (Shareef et al., 2016)
3	2-Cyclohexylpiperidine	Alkaloid (Heterocyclic organic compound)	Antimicrobial and anti-inflammatory properties (Lincy et al., 2015).
4	1-Heptatriacotanol	Alcohol	Antioxidant, anti-inflammatory, hypocholesterolemic, antimicrobial and anticancer properties (Shareef et al., 2016; Igwe and Okwu 2013; Junwei et al., 2018; Kalaimagal, 2019; Abdulhafiz et al., 2020).
5	17-Pentatriacontene	Unsaturated aliphatic hydrocarbon	Anti-hypercholesterolemic properties (Abdulhafiz et al., 2019)
6	Phytol, acetate	Acyclic diterpenoids	cancer-preventive, antimicrobial, anti-inflammatory, diuretic, antioxidant properties (Vandana et al., 2018; Godara et al., 2019)
7	3,7,11,15-Tetramethyl-2-hexadecen-1-ol	Phytol	antinociceptive, antioxidant and anti-inflammatory properties (Selvi et al., 2017)
8	Pentadecanoic acid, 13-methyl-, methyl ester	Fatty acid ester	antibacterial and antifungal activities (Ghazala et al., 2004).
9	Hexadecanoic acid, 1 - (hydroxymethyl)-1,2-ethanediyl ester	Fatty acid ester	antioxidant, hypocholesterolemic, antiandrogenic, hemolytic, α- reductase inhibitor activity (Arora and Kumar 2017)
10	9,12,15-Octadecatrienoic acid, 2-phenyl-1,3-dioxan-5-yl ester	Esther	antimicrobial and anti-inflammatory properties (Kamel et al., 2022)
11	Diisooctyl phthalate	Benzoic acid ester	antimicrobial and antifouling properties (Ingole, 2016)
12	Squalene	Triterpene (Lipid)	Antioxidant and antitumor properties (Igwe and Okwu, 2013)

## Discussion

According to earlier studies (Clarke and Clarke, 1977; Kalu et al., 2016), any substance that has an LD_50_ value of 1000 mg/kg.BW and above is considered to be safe for consumption. Therefore, the LD_50_ for this plant as reported in this study might suggest its non-toxicity.

Change in body weight is a parameter of enormous significance in the assessment of first indicators of toxicity (Enegide et al., 2013; Uzor et al., 2014; Emelike et al., 2020; Achi et al., 2024). In this study, administration of CPH to the rats decreased their weight gain and body weights relative to the control. This decrease could have arisen from the mobilization and utilization of endogenous proteins during the metabolism of CPH, thereby reducing the quality of proteins available to promote growth process of the body (Eleazu et al., 2014b; Udeme et al., 2015). Increased body weights and weight gain following administration of *J. secunda* to the rats in study groups 1 and 2, suggests mitigation of CPH-instigated weight loss in rats. 

The liver and kidney are important organs that are involved in drug metabolism and they are greatly susceptible to drug-associated toxicities because of their structure, roles in drug metabolism and anatomic position (Abarikwu et al., 2017). Moreover, the prevalence of liver and kidney injuries that come from intake of chemotherapeutic drugs has continued to soar, thereby limiting the success rates of these chemotherapeutic agents in clinical practice. CPH is a widely used chemotherapeutic agent that is used to treat different types of tumor. In spite of its demonstrated efficacy as a chemotherapeutic agent, the hepatotoxicity and nephrotoxicity that arise from its usage is limiting its therapeutic usage (Caglayan et al., 2018). 

Organ weight is reported to be one of the most sensitive and reliable indicators of the toxicity of a substance. This is due to the fact that notable changes in the weights of organs between treated and untreated animals could occur in the absence of any change in morphology or they may occur before any change in morphology (Kalu et al., 2016; Emelike et al., 2020; Balogun et al., 2014; Marcela et al., 2017). In addition, liver and kidney functions can be measured by the serum levels of different molecules or the activities different enzymes (Emelike et al., 2020; Udeme et al., 2015). Therefore, in this study, the effect of methanolic extract of *Justicia secunda* against CPH-instigated hepatic and renal toxicities in rats was determined by measurement of organ weights (the liver and kidney), the liver function markers (AST and ALT) and the kidney function markers (urea and creatinine) in the sera of the rats. 

In this study, CPH administration decreased the absolute liver and kidney weights and the relative weights of the liver and kidney of the rats, confirming the hepatic and renal toxicities of this drug as reported in several studies (Moghe et al., 2015; Raza and Alghasham, 2011; Habibi et al., 2015). Following administration of *J. secunda*, the increased absolute weights of the liver and kidney as well as the increased relative weights of the liver and kidney of the rats in study groups 1 and 2, might indicate the ability of *J. secunda *to mitigate CPH-instigated liver and kidney toxicities in rats. 

AST catalyzes an α-amino group transfer from aspartate to α-ketoglutarate thereby forming oxaloacetate and glutamate whereas ALT catalyses α-amino group transfer from alanine to α-ketoglutarate thereby forming glutamate and pyruvate (Eleazu et al., 2019). Whereas AST can be found in the heart, liver, skeletal muscle and red blood cells, ALT is found mainly in the liver (Shivananda, 2007). When there is damage to the liver cells or disease of the liver, serum activities of AST and ALT become raised (Shivananda, 2007; Vasudevan et al., 2013; Lala et al., 2021). 

The current study found an elevation in the serum AST and ALT activities in the CYP group further affirming that CPH induced hepatic toxicity to the rats. We suspect that these enzymes may have leaked from the liver cytosol into the circulation due to CPH-induced hepatic toxicity. 

Interestingly, administration of *J. secunda *to the rats in study groups 1 and 2, decreased the activities of these diagnostic enzymes in the sera of the rats, indicating the potentials of *J. secunda *in mitigating CPH-instigated liver injury. 

Formation of urea occurs in the liver since it is derived from the metabolism of proteins and it is subsequently removed from the blood through the kidneys by filtration (Abraham and Isaac, 2011; Shahrbaf and Assadi, 2015). During renal disease or impairment, the rate of removal of urea by the kidneys will be altered, leading to increased levels of urea in the blood (Emelike et al., 2020). Creatinine is a waste product that is obtained from metabolism of creatine phosphate and it is eliminated from the body through glomerular filtration and proximal tubular secretion in the kidney. However, when there is renal impairment or disease, defective filtration of creatinine by the kidneys occurs, leading to increased concentrations of creatinine in the blood (Emelike et al., 2020). 

Our study found elevated serum concentrations of urea and creatinine in the CYP group, also affirming the renal toxicity of CPH. Similar reports on elevation of serum urea and creatinine in rats induced with CPH were also given by Shabana et al. (2012). 

*J. secunda *demonstrated the capacity to protect the kidneys against CPH-instigated toxicity and this was evident from the decrease in the serum urea and creatinine concentrations of the rats in study groups 1 and 2. 

It has been reported that bioactivation of CPH triggers the generation of ROS which attack both healthy and cancerous cells, leading to increased oxidative stress and adverse effects (Kocahan et al., 2017). In this study, exposure of the rats to CPH overwhelmed the antioxidant defense systems in their liver and kidney tissues, as was evident from the decreased activities and concentrations of the antioxidant markers- SOD, CAT, GST, GPx and GSH but increased concentration of MDA in their liver and kidney tissues.

Interestingly, *J. secunda *demonstrated protective action against CPH-instigated hepatic and renal oxidative stress, as was apparent from the increased activities of SOD, CAT, GST and GPx, as well as the increased concentration of GSH but decreased concentration of MDA in the liver and kidney tissues of the rats in study groups 1 and 2. 

The protective action of *J. secunda* against hepatic and renal injury as demonstrated in this study, might have come from the antioxidant property of *J. secunda* and its scavenging ability against the toxic metabolite that was generated during CPH activation in the rats’ liver and kidney. The current study therefore reveals that *J. secunda* mitigates CPH- instigated liver and kidney injuries by arresting oxidative stress and enhancing antioxidant defenses. 

Our study further showed reduced concentration of total protein in the liver and kidney of the CYP group. The elevation in the hepatic and renal concentrations of total proteins of the rats in study groups 1 and 2 could be attributed to the attenuation of hepatic and renal oxidative stress and injury by *J. secunda *due to its antioxidant activity. 

GC-MS has been demonstrated to be a reliable technique for identifying the components of volatile compounds, hydrocarbons, alcoholic acids, esters and others (Eleazu and Eleazu, 2015). The compounds in the extract that their biological properties could be identified were as follows: p-Xylene which added up to 2.351% of the extract was suggested to possess anti-cancer activity (Manzoor et al., 2016). 7-epi-cis-sesquisabinene hydrate that accounted for 0.779% of the extract was suggested to have anti-cancer activity (Shareef et al., 2016). 2-Cyclohexylpiperidine which accounted for 1.199% of the extract was earlier stated to have antimicrobial and anti-inflammatory activities (Lincy et al., 2015). 1-Heptatriacotanol which accounted for 0.79% of the extract was stated to have antioxidant, anti-inflammatory, hypercholesterolemic, antimicrobial, and anticancer properties (Ganesh and Mohankumar, 2017; Shareef et al., 2016; Igwe and Okwu, 2013; Junwei et al., 2018; Kalaimagal, 2019; Abdulhafiz et al., 2020). 17-Pentatriacontene which constituted 1.241% of the extract was stated to have antiseptic properties (Igwe and Okwu, 2013). Phytol acetate which constituted 13.179% of the extract was earlier stated to have cancer-preventive, antimicrobial, anti-inflammatory, diuretic and antioxidant activities (Abdulhafiz et al., 2019; Vandana et al., 2018). 

3,7,11,15-Tetramethyl-2-hexadecen-1-ol which constituted 6.979% of the extract was stated to have antinociceptive, antioxidant, and anti-inflammatory actions (Godara et al., 2019). Pentadecanoic acid, 13-methyl-, methyl ester that constituted 3.238% of the extract was reported to possess antibacterial and antifungal activities (Selvi et al., 2017). Hexadecanoic acid, 1 - (hydroxymethyl)-1,2-ethanediol ester, a fatty acid ethyl ester that constituted 3.037% of the extract was reported to possess antioxidant, hypocholesterolemic, antiandrogenic, hemolytic and alpha reductase inhibitor activity (Ghazala et al., 2004). 9,12,15-Octadecatrienoic acid, 2-phenyl-1,3-dioxan-5-yl ester which constituted 3.907% of the extract was stated to have antimicrobial and anti-inflammatory properties (Arora and Kumar, 2017). Diisooctyl phthalate which accounted for 5.381% of the extract was stated to have antimicrobial and antifouling properties (Kamel et al., 2022) while squalene which constituted 9% of the extract was stated to have antioxidant and antitumor properties (Ganesh and Mohankumar, 2017; Igwe and Okwu, 2013; Ingole, 2016). These compounds with identified pharmacological activities made up 51.08% of the extract while the compounds that made up 48.92% either had unknown biological activities or their identities were unknown. 

The plethora of compounds in the *J. secunda *extract with antioxidant properties may have contributed to the antioxidant properties of *J. secunda *and its protective action against CPH-induced hepatic and renal toxicities as obtained in this study.

The key limitation of our current study is that we could not determine the histology of the liver and kidneys of the CPH-induced rats that were treated with methanol extract of *J. secunda*. However, the strength of this article lies in our determination of the oxidative stress markers in the liver and kidneys of the rats, which provided a clue to the rationale for the protective action of *J. secunda *against CPH -induced hepatic and renal toxicities.

In conclusion, the present study showed the protective action of *J. secunda *and its protective action against CPH-induced hepatic and renal toxicities which could be attributed to the antioxidant property of *J. secunda* and its scavenging ability. Finally, this study revealed that *J. secunda* contains a plethora of bioactive compounds that support its antioxidant properties and protective actions against CPH-induced hepatic and renal toxicities in rats.
